# ChatGPT-polished writing boosts the risk of human-authored manuscripts being miscredited as AI-generated

**DOI:** 10.1016/j.jdin.2025.05.013

**Published:** 2025-06-19

**Authors:** Jinge Wang, Neil Jairath, Evelyn Shue, Peter L. Perrotta, Gangqing Hu

**Affiliations:** a Department of Microbiology, Immunology & Cell Biology, West Virginia University, Morgantown, West Virginia; b The Ronald O. Perelman Department of Dermatology, New York University Grossman School of Medicine, New York, New York; c Department of Biology, Duke University, Durham, North Carolina; d Department of Pathology, Anatomy, and Laboratory Medicine, West Virginia University, Morgantown, West Virginia

**Keywords:** AI, artificial intelligence, ChatGPT, epidemiology, ethics, fairness, large language models, manuscript editing, medical education, machine learning

*To the Editor:* Large language model chatbots such as ChatGPT are valuable tools for medical writing.^1^^,^^2^ Concurrently, GPT detectors like GPTZero have emerged to safeguard content authenticity. Here, we addressed 2 key questions in dermatology manuscript writing: (1) What are the trends in AI-assisted writing for dermatology manuscripts, particularly among non-native English authors? (2) To what extent could manuscripts with original contributions, but polished by ChatGPT, be miscredited as AI-generated?

We analyzed *Research Letters* published in JAAD in 2020 and 2024. Authors from non-English-speaking countries (China, Korea, and Japan) were compared with U.S. authors, representing English-speaking countries. We identified 58 letters from the former group and randomly selected 2 or 3 letters per issue from the latter to match the sample size. The main text of each letter was evaluated by GPTZero to estimate an AI-generated probability. Given the surge in chatbot usage since December 2022, we hypothesized a higher probability of AI-assisted text in 2024 than in 2020. Comparison of the letters between 2020 and 2024 confirmed this trend for non-native English authors (Fig 1). In contrast, no significant difference was observed for the U.S. authors.

**Fig 1:**
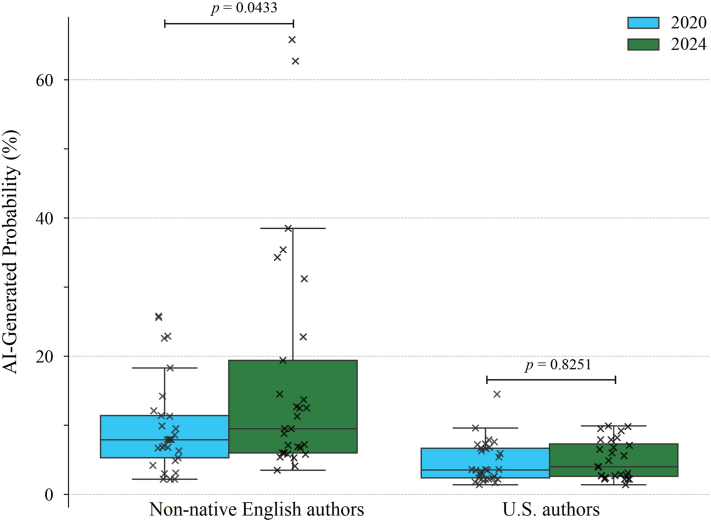
Trend of AI-assisted writing among non-native English authors and U.S. authors in dermatology publications. Box plots showing AI-generated probabilities assigned by GPTZero for research letters published in *JAAD* in 2020 and 2024, grouped by non-native English authors and U.S. authors. *P* values by *t*-test.

Building on this finding, we then investigated how ChatGPT-assisted polishing affects the likelihood of text being flagged as AI-generated. Using Application Programming Interface (version “chatgpt-4o-latest”, accessed December 2024), we prompted ChatGPT to *“enhance the readability and flow”* while preserving *“original meaning and intent”* for all letters. Both original and polished versions were evaluated by GPTZero. While 97% to 100% of the original letters were classified as human-generated, 75% to 85% of the polished versions were flagged as AI-generated, with 15% to 25% labeled at high confidence (Fig 2); Similar results were obtained with Originality.AI. A focused review of 10 letters flagged by GPTZero as 100% AI-generated after polishing revealed alterations in meaning for on average 2 to 3 sentences per letter, emphasizing the necessity of careful human proofreading postpolishing.

**Fig 2:**
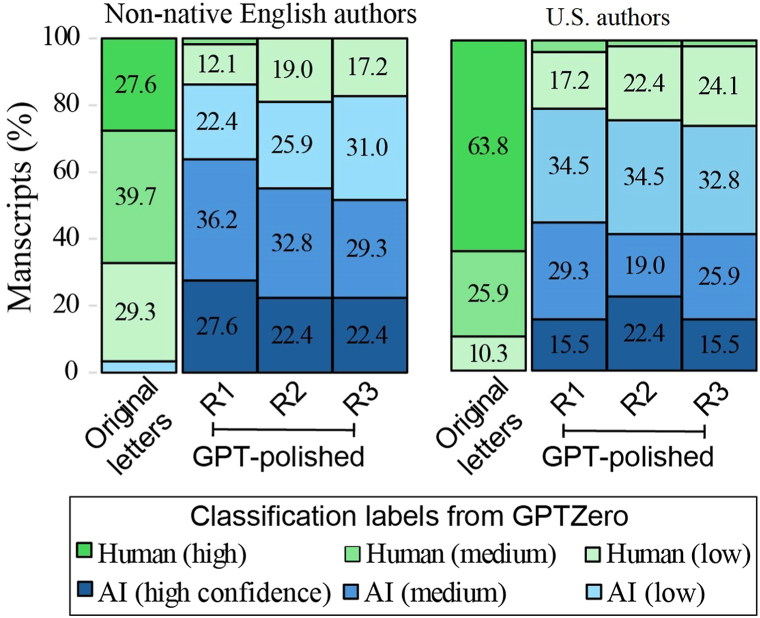
Increased risk of manuscripts being miscredited as AI-generated after being polished by ChatGPT. Distribution of orignal letters and GPT-polished versions (in triplicates: “R1”, “R2”, and ‘R3″) labeled by GPTZero as human-generated or AI-generated with varying levels of confidence (low, medium, and high). Papers were grouped by non-native English authors and U.S. authors, combining 2020 and 2024.

This study has limitations. The simulated polishing was applied to papers already subjected to rigorous peer review and professional editing, potentially limiting ChatGPT’s impact on readability enhancement. Additionally, the evaluation did not consider afterwards human proofreading. Moreover, GPTZero cannot detect data fabrication. Nevertheless, our findings raise a critical concern: ChatGPT-assisted polishing can substantially increase the risk of original contributions being miscredited as AI-generated. Advanced tools capable of distinguishing human-authored, AI-polished from unethical, AI-generated text are urgently needed.^3^ Such tools should promote fairness, particularly for non-native English authors^1^^,^^2^ and the emerging Generation Z,^4^ who are increasingly likely to utilize AI-assistance for high-stakes writing tasks like manuscript preparation and medical school applications.

While we do not aim to advocate for AI-assisted writing, publishers and other stakeholders adopting GPT detectors should be aware of their current limitations. For authors, transparent disclosure of AI assistance in writing, including disclosing chat history when appropriate, help mitigate the risks of human contribution being miscredited by using current GPT detectors.

Prompts, an example letter,^5^ GPTZero and Originality.AI scores, and additional results are available through Mendeley (Supplementary Tables I-III and Figs 1 to 4, available via Mendeley at https://data.mendeley.com/datasets/j74rcf56fy/3).

## Declaration of generative AI and AI-assisted technologies in the writing process

During the preparation of this work, G.H. used ChatGPT-4o to improve readability, clarity, and professionalism in writing through multiple rounds of iteration. After using this tool, all authors reviewed and edited the content as needed and take full responsibility for the content of the publication.

## Conflicts of interest

None disclosed.
